# Automated prediction of early spontaneous miscarriage based on the analyzing ultrasonographic gestational sac imaging by the convolutional neural network: a case-control and cohort study

**DOI:** 10.1186/s12884-022-04936-0

**Published:** 2022-08-05

**Authors:** Yu Wang, Qixin Zhang, Chenghuan Yin, Lizhu Chen, Zeyu Yang, Shanshan Jia, Xue Sun, Yuzuo Bai, Fangfang Han, Zhengwei Yuan

**Affiliations:** 1 Key Laboratory of Health Ministry for Congenital Malformation, Shengjing Hospital, China Medical University, No. 36, Sanhao Street, Heping District, Shenyang, 110004 P.R. China; 2 Department of Ultrasound, Shengjing Hospital, China Medical University, Shenyang, 110004 P.R. China; 3 College of Medicine and Biological Information Engineering, Northeastern University, Shenyang, Liaoning 110819 P. R. China; 4 Department of Pediatric Surgery, Shengjing Hospital, China Medical University, Shenyang, 110004 P.R. China; 5 College of Medical Information Engineering, Guangdong Pharmaceutical University, Guangzhou, Guangdong 510006 P.R. China

**Keywords:** Early pregnancy, Spontaneous abortion, Sonographic, Deep learning

## Abstract

**Background:**

It is challenging to predict the outcome of the pregnancy when fetal heart activity is detected in early pregnancy. However, an accurate prediction is of importance for obstetricians as it helps to provide appropriate consultancy and determine the frequency of ultrasound examinations. The purpose of this study was to investigate the role of the convolutional neural network (CNN) in the prediction of spontaneous miscarriage risk through the analysis of early ultrasound gestational sac images.

**Methods:**

A total of 2196 ultrasound images from 1098 women with early singleton pregnancies of gestational age between 6 and 8 weeks were used for training a CNN for the prediction of the miscarriage in the retrospective study. The patients who had positive fetal cardiac activity on their first ultrasound but then experienced a miscarriage were enrolled. The control group was randomly selected in the same database from the fetuses confirmed to be normal during follow-up. Diagnostic performance of the algorithm was validated and tested in two separate test sets of 136 patients with 272 images, respectively. Performance in prediction of the miscarriage was compared between the CNN and the manual measurement of ultrasound characteristics in the prospective study.

**Results:**

The accuracy of the predictive model was 80.32% and 78.1% in the retrospective and prospective study, respectively. The area under the receiver operating characteristic curve (AUC) for classification was 0.857 (95% confidence interval [CI], 0.793–0.922) in the retrospective study and 0.885 (95%CI, 0.846–0.925) in the prospective study, respectively. Correspondingly, the predictive power of the CNN was higher compared with manual ultrasound characteristics, for which the AUCs of the crown-rump length combined with fetal heart rate was 0.687 (95%CI, 0.587–0.775).

**Conclusions:**

The CNN model showed high accuracy for predicting miscarriage through the analysis of early pregnancy ultrasound images and achieved better performance than that of manual measurement.

**Supplementary Information:**

The online version contains supplementary material available at 10.1186/s12884-022-04936-0.

## Background

Miscarriage is the most common early pregnancy complication affecting about 30% of pregnancies following assisted reproduction and 10% of spontaneously conceived pregnancies [1–3]. Even after the embryonic cardiac activity is detected, the subsequent rate of miscarriage remains 5.2 to 10.4% [4, 5]. Miscarriages very often have a significant social and psychological effect on the mother, especially for pregnant women who have received assisted reproduction or experienced repeated abortion [6, 7]. Therefore, for early pregnancies with fetal heart activity, the development of a prediction model for miscarriage can not only help obstetricians decide the frequency of subsequent ultrasound examinations but also alleviate the great psychological pressure and anxiety of the pregnant women due to fear of miscarriage.

Several studies have demonstrated that sonographic findings in early pregnancy have prognostic value in predicting pregnancy outcomes. Various signs have been described, such as mean gestational sac diameter [8], crown-rump length [9, 10], fetal heart rate [11–13], abnormal sonographic appearance of the yolk sac [14], etc. The examination of multiple sonographic features may improve the ability to predict subsequent pregnancy loss, however, their calculations are somewhat complicated, which limited ease of clinical implementation.

Deep learning algorithms have been widely applied in the field of image diagnosis and prediction owing to their advantages of being fast, accurate, and reproducible [15]. One of the most powerful deep learning approaches related to images is convolutional neural networks (CNNs). Recent research on medical data has shown that deep CNNs can be successfully used to extract and analyze information obtained from medical images [16–18]. In fetal ultrasonography (US), CNNs have shown potential for standard plane recognition, detection, and localization [19]. Training a CNN algorithm to analyze early pregnancy US images and predict the spontaneous miscarriage risk, would be a more convenient alternative for assessing prognosis. However, to our knowledge, there have been no reports to date on the prediction of miscarriage by deep learning.

In this study, we presented a novel procedure using the deep learning algorithms to predict the spontaneous miscarriage risk after the appearance of embryonic cardiac activity in 6 to 8 weeks through US images of the gestational sac.

## Methods

### Study design and patient population

**Fig. 1:**
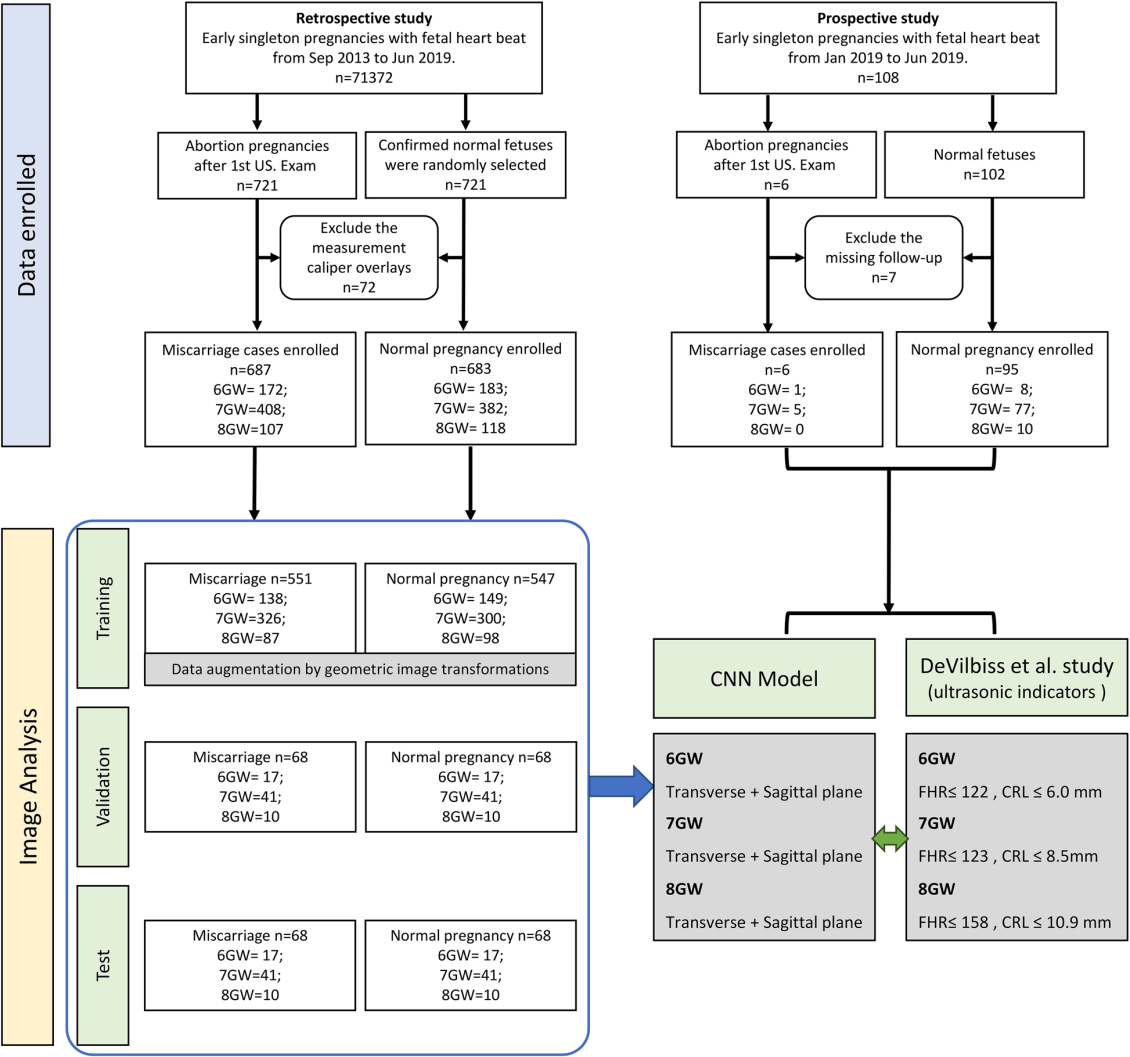
Data enrollment in the present study. This figure is a flowchart showing the strategies by which we searched for the database to enroll the study population. GW: gestational week; CNN: convolutional neural networks; FHR: fetal heart rate; CRL: crown-rump length; US: ultrasound

This study was approved by the ethics committee of the Shengjing Hospital of China Medical University (2016PS243K) and was performed in accordance with the Declaration of Helsinki. The fully anonymized ultrasound images in the database were used in the study, and a CNN was constructed by training, validation, and test data sets (Fig. 1).

The retrospective review was conducted from September 2013 to June 2019, using information from the database of Shengjing Hospital of China Medical University. A total of 71,372 patients with early singleton pregnancies of gestational age between 6 and 8 weeks showing embryonic cardiac activity were retrospectively analyzed. There were 721 patients identified who had positive fetal cardiac activity on their first ultrasound but then experienced a miscarriage. Besides, 721 confirmed normal fetuses were randomly selected from the same database to serve as normal controls. A total of 72 cases were excluded because of measurement caliper overlays which affected the effectiveness of machine learning (34 cases of miscarriage, 38 normal fetuses). In order to better verify the predictive ability of the model, we also conducted a prospective study from January 2019 to June 2019, of 108 women with singleton pregnancies (6 of whom experienced miscarriages and 102 of whom carried the pregnancy to term). Seven patients were excluded because of missed follow-up. The relevant clinical information of the enrolled patients is summarized in Table 1. The gestational age of the enrolled patients at the time of the ultrasound was calculated based on the difference between the date of the ultrasound and the last menstrual period.

**Table 1:** Demographic and pregnancy characteristics of women included in the study

Parameter	Retrospective study			Prospective study		
	Live birth (*n* = 683)	Miscarriage(*n* = 687)	*P*	Live birth (*n* = 95)	Miscarriage (*n* = 6)	*P*
Maternal age in years^a^	33.0(19–42)	32.0(23–45)	0.48	30.0(24–36)	32.0(25–39)	0.52
GA in weeks^a^	6(6–8)	6(6–8)	0.62	7(6–8)	7(6–7)	0.58
Gravidity^a^	2.0(1–4)	3.0(1–6)	0.57	1.0(1–3)	2.0(1–4)	0.60
History of RPL (%)^b^	57(8.3)	36(5.2)	0.42	6(6.3)	0(0.0)	1.00
IVF or ICSI (%)^b^	178(26.1)	145(21.1)	0.38	13(13.7)	1(16.7)	1.00
GA at delivery or miscarriage (weeks)^a^	39 + 4(34 + 6 to 42 + 0)	9 + 3(7 + 0 to 16 + 2)	NA	39 + 2(37 + 0 to 41 + 5)	10 + 3(8 + 5 to 15 + 6)	NA

*GA* gestational age, *ICSI* intracytoplasmic sperm injection, *IVF* in-vitro fertilization, *NA* not applicable, *RPL* recurrent pregnancy loss

^a^Data are given as median (range)

^b^Data are given as number (percent)

### Data preparation for automated image analysis

US images in the retrospective study were extracted from the hospital ultrasound workstation. The ultrasound examination was conducted using Voluson 730 Expert (GE Medical Systems, Zipf, Austria), Affiniti 70 ultrasound systems (Philips Medical Systems, Amsterdam, the Netherlands), and Aixplorer® (SuperSonic Imagine, France). Images were taken using a transvaginal transducer with a frequency range from 3 to 9 MHz, and the center frequency of the transvaginal transducer was 6.5 MHz. All scans were performed by accredited sonographers with 3–15 years (mean: 8 years) experience. In the prospective study, ultrasound examination was conducted using Affiniti 70 ultrasound systems (Philips Medical Systems, Amsterdam, the Netherlands) equipped with a 3–9 MHz transvaginal transducer. All scans were performed by accredited sonographers with 5 to 15 years’ experience. Crown-rump length and fetal heart rate were measured.

In all the cases, the images were acquired by an experienced sonographer according to a unified acquisition process in the routine ultrasound examination. The specific process was as follows: on the sagittal section of the uterus, display the largest sagittal section of the gestational sac clearly, and save the image; on the transverse section of the uterus, the largest transverse section of the gestational sac was clearly displayed, and the image was preserved. The images in the median sagittal plane and the perpendicular transverse plane were selected for further analysis. All US images included were in JPEG format and 768 × 1024 pixels.

### Gestational sac region segmentation

**Fig. 2:**
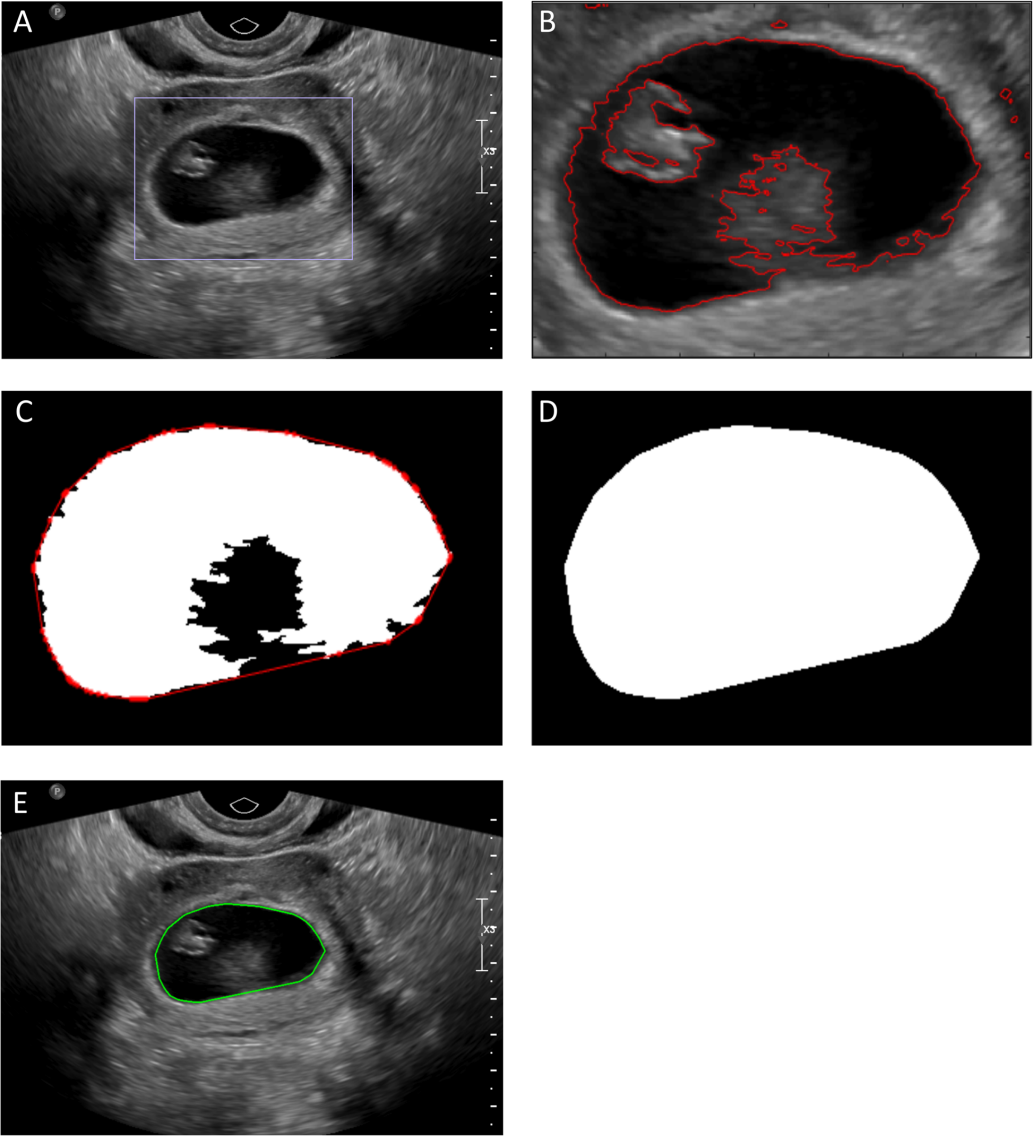
The segmentation process of the gestational sac. **A** Firstly, the region of interests (ROI) was selected which contained the complete gestational sac. **B** Secondly, the region of gestational sac was segmented using level set method. **C** Thirdly, the largest connected area was found. **D** Fill the largest connected area. **E** Finally, the complete edge of the gestational sac was extracted and marked on the original image

In order to avoid the interference of the grayscale information of the myometrium around the gestational sac to the CNN learning results, we first segmented the gestational sac region [20]. For all ultrasound images, the region of interest containing the complete gestational sac was selected for preprocessing (Fig. 2A). A level set was used to segment the region of interest, and then the smallest convex polygon with the largest connected region was found and filled (Fig. 2B, C, D). The complete edge of the gestational sac was extracted and marked on the original image (Fig. 2E).

**Fig. 3:**
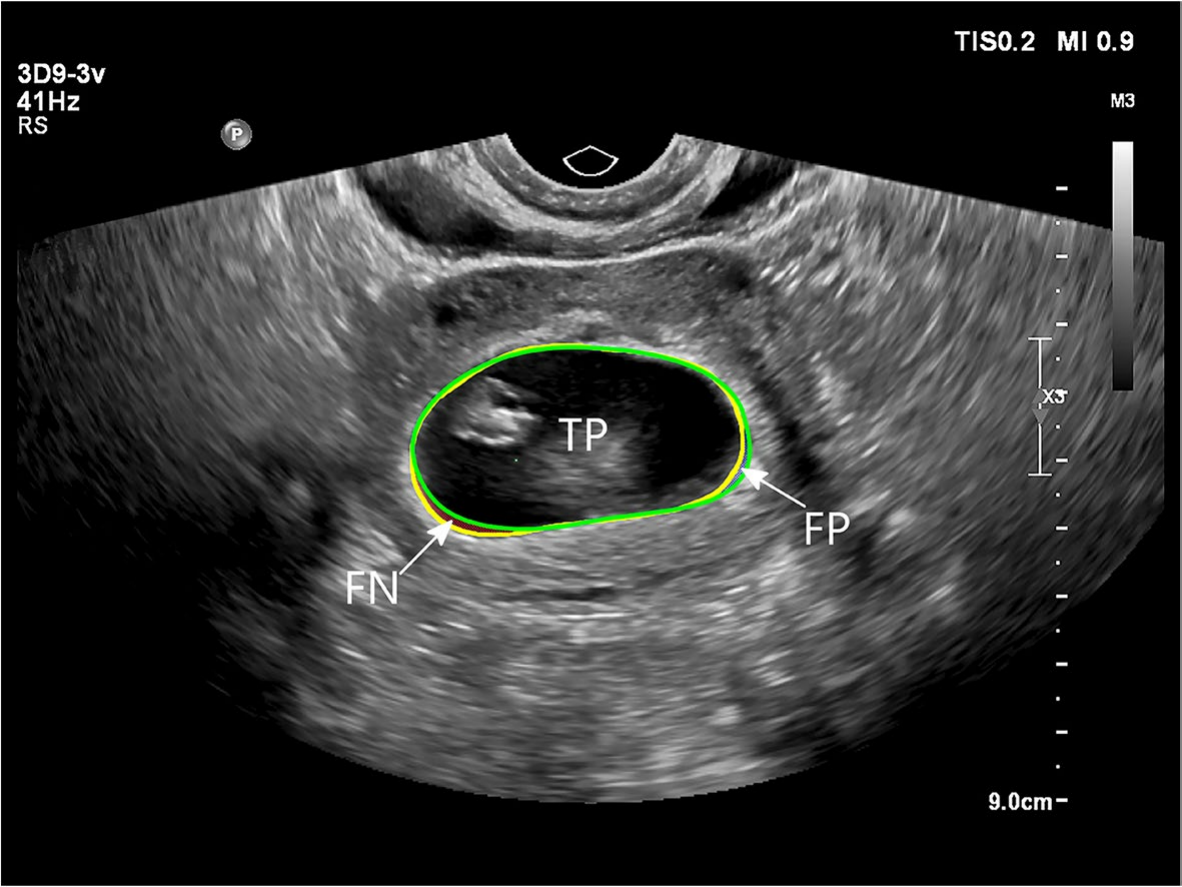
Illustration of the quantitative accuracy assessment of gestational sac region segmentation. The yellow ellipse is an area (G) manually labeled by a doctor before the test, while the green ellipse indicates the segmentation area (S) predicted by the algorithm. The intersection area between S and G is the true positive predicted area (TP). FP (false positive predicted area) = S-TP; FN (false negative predicted area) = G-TP

To quantitatively assess the accuracy of the segmentation, we defined the segmentation area as “S”, and two experienced sonographers were trained and asked to manually trace along the inner edges of the trophoblast together, which we used as the “gold standard” (GT). The intersection area between S and GT served as the true positive predicted area (TP); S minus the TP served as the false positive predicted area (FP), and GT minus TP equaled the false-negative predicted area (FN). We calculated areas by counting the number of pixels, as illustrated in Fig. 3.

Performance of segmentation was assessed using precision, recall, and the Dice coefficient (DICE), which were calculated to measure the extent of overlap between human-labeled and machine-segmented regions. The values of these metrics all ranged from 0 to 1, with larger values representing better performance [19].

They are defined as follows:

- Precision = TP / (TP + FP),
- Recall = TP / (TP + FN),
- DICE = 2 × TP / (S + GT).

### Convolutional neural network

**Fig. 4:**
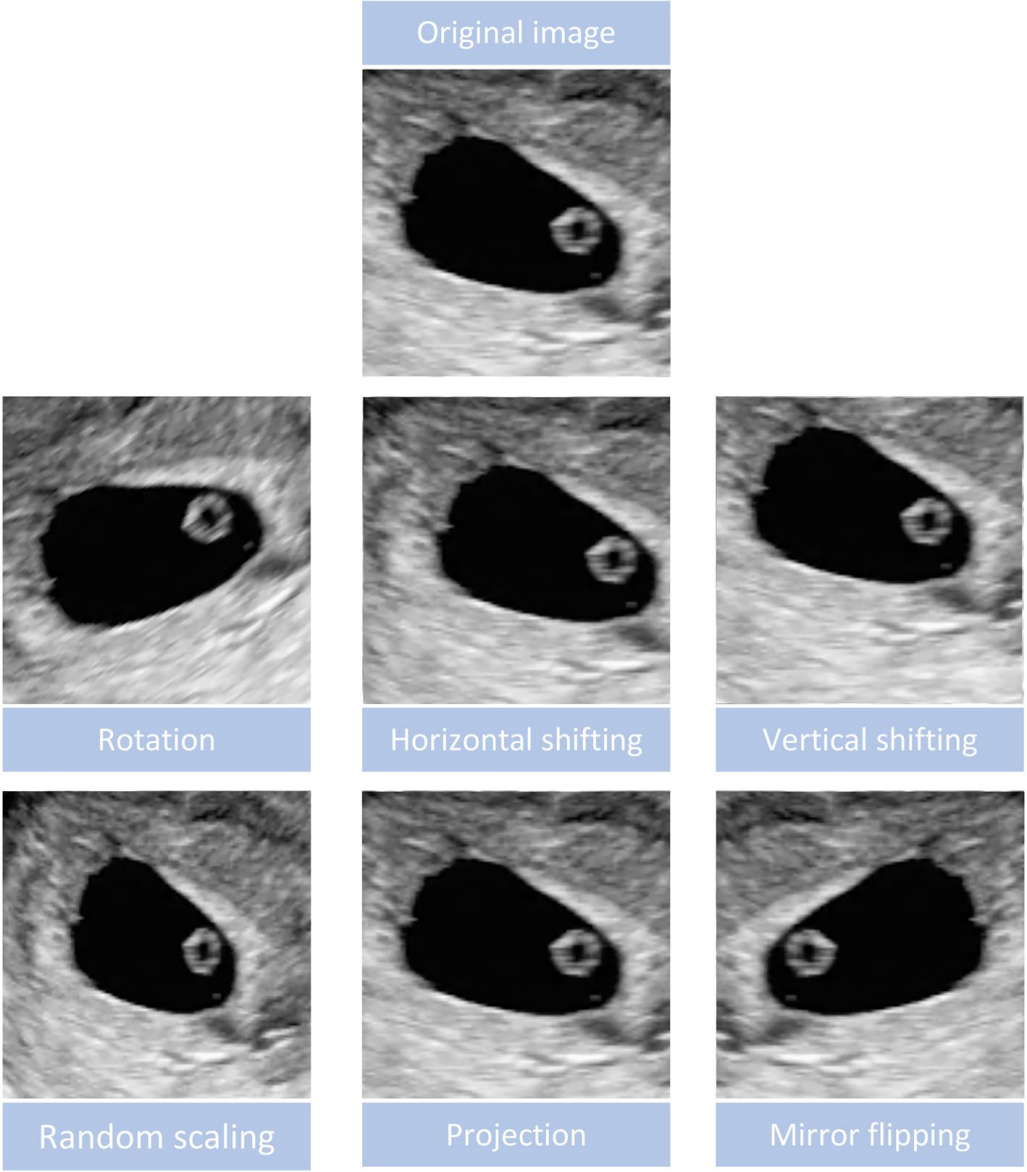
Data augmentation was performed by flipping, rotation, scaling, shifting and projection

Segmented pregnancy sacs were used for CNN learning. Data augmentation was used to increase training data size and to help the trained model cope with variabilities that were not in the original training set. In this case, data augmentation was performed through random geometric image transformations with random parameters, including rotation (from − 40° to 40°), scaling (±20% of the original image sizes), horizontal and vertical shifting (smaller than 20% of the input image sizes for each direction), projection and mirror flipping (on horizontal and vertical directions) (Fig. 4). When there are blank areas after transformations such as shifting and rotation, we randomly used one type of filling methods (namely ‘constant’, ‘nearest’, ‘reflect’ and ‘wrap’). The data augmentation strategy has proved to help prevent network overfitting and memorization of the exact training image details [21]. The training group images were input into the CNN-based VGG19 model [22]. The programs of VGG19 used in our experiments can be downloaded from a public resource on a much popular program platform named ‘github’. The website is https://github.com/fchollet/deep-learning-models/releases/download/v0.1/vgg19_weights_tf_dim_ordering_tf_kernels_notop.h5. More composition details of the above architecture are shown in https://github.com/fchollet/deep-learning-models. And the programs of how to use VGG19 model by Keras API and the environment setting are shown as supplemental materials.

**Fig. 5:**
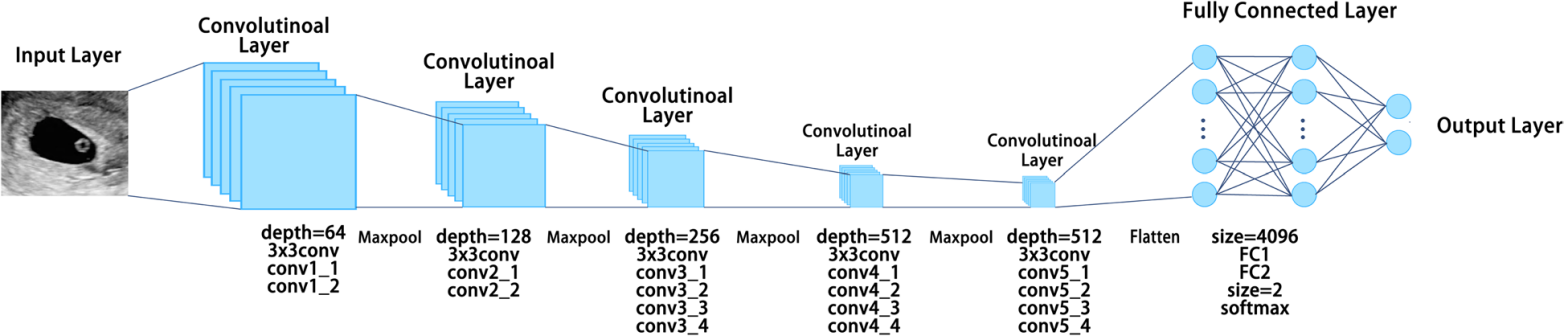
A schematic illustration of the convolutional neural network of VGG19

VGG19 is one type of VGG models containing the most convolutional layers. ‘19’ means 16 convolutional layers and 3 fully-connected layers (Fig. S1). The convolutional layers are responsible for extracting high-order features. And the fully-connected layers are used for features projection and classification [22]. Workflow of the VGG19 models for automated prediction of miscarriage is shown in Fig. 5. The classifier was used to classify the gestational sac features in the ultrasound images captured by the CNN for the final classification of miscarriage and ongoing pregnancy.

In the evaluation step, 10-fold cross-validation was used. A total of 1/10 of the positive and negative samples (136 cases) before the expansion was selected as the test set, 1/10 of the samples (136 cases) was chosen as the validation set, and the remaining 4/5 samples (1098 cases) were used as the training set. Ultrasound images from the prospective study were used as test sets to verify the predictive performance of the model. The whole testing procedure and computer programming environment are similar to our prior work on lung cancer prediction shown in [23].

### The manual ultrasound characteristics

As prenatal US has become a standard element of routine prenatal care [24], many studies have focused on the ability of early ultrasound characteristics, such as fetal heart rate and crown-rump length to predict miscarriage. In order to compare the predictive power of different methods, we analyzed the fetal heart rate and crown-rump length according to the research of DeVilbiss et al. [5] using our prospective data. Low fetal heart rate (≤122, 123, and 158 bpm) and small crown-rump length (≤6.0, 8.5, and 10.9 mm) for gestational weeks 6, 7, and 8, respectively, were used as independent predictors of clinical pregnancy loss.

### Statistical analyses

The Mann-Whitney *U* test or χ^2^ test was used for comparing maternal and embryonic characteristics between the group of pregnant women with spontaneous miscarriage and the group of pregnant women who carried their pregnancies to full term. The diagnostic indicators of CNN (sensitivity, specificity, accuracy, positive predictive value (PPV), and negative predictive value (NPV)) were calculated. The receiver operating characteristic (ROC) analysis was performed using MedCalc, version 18.11 (MedCalc Software Ltd., Ostend, Belgium), and the diagnostic index was calculated using SPSS, version 20.0 (IBM Corp, Armonk, NY, United States). A *P*-value < .05 was considered statistically significant.

## Results

### Gestational sac region segmentation

The total set of 2942 images (the transverse and sagittal images of the 1370 retrospective cases and 101 prospective cases) of normal and abnormal cases were used for training, validation, and testing of the gestational sac region segmentation. The average DICE index was 90.69%, the recall was 94.56%, and the precision was 87.86%, indicating that the gestational sac could be well segmented automatically.

### Classification

#### Diagnostic efficiency of the deep learning model

In the retrospective study, the deep learning model achieved good performance in predicting miscarriage with the use of early pregnancy ultrasound images. The overall accuracy was 80.32%, and the overall sensitivity, specificity, PPV, and NPV for prediction of miscarriage were 80.73, 80.91, 81.18, and 80.02%, respectively (Table 2).

**Table 2:** Classification results of different gestational weeks and different planes

Gestational age	Sonographic planes	Accuracy,% (95%CI)	Sensitivity,% (95%CI)	Specificity,% (95%CI)	PPV,% (95%CI)	NPV,% (95%CI)	AUC (95%CI)
**sixth week**	Tran	83.07 (78.10, 93.40)	82.30 (72.45, 95.34)	83.89 (73.74, 94.04)	84.08 (72.83, 95.34)	81.87 (71.08, 92.67)	0.863 (0.737, 0.922)
	Sag	82.59 (74.16, 94.76)	82.39 (68.60, 98.10)	82.79 (71.90, 94.90)	82.96 (67.94, 97.97)	82.29 (72.05, 92.53)	0.849 (0.716, 0.935)
	Tran+ Sag	86.53 (83.11, 97.83)	88.71 (79.80, 97.99)	83.57 (73.66, 94.08)	81.07 (70.61, 91.53)	88.85 (78.50, 99.19)	0.904 (0.781, 0.950)
**seventh week**	Tran	76.94 (76.08, 89.17)	72.44 (65.19, 80.16)	80.94 (71.14, 91.54)	83.96 (73.46, 94.47)	68.05 (55.67, 94.47)	0.827 (0.706, 0.833)
	Sag	78.52 (76.88, 88.62)	76.62 (69.34, 84.53)	81.42 (72.99, 90.67)	82.93 (72.30, 93.55)	74.73 (63.17, 86.28)	0.829 (0.733, 0.838)
	Tran+ Sag	78.35 (76.19, 91.90)	77.96 (64.83, 91.90)	78.87 (69.46, 89.49)	79.26 (65.54, 92.99)	77.61 (67.90, 87.33)	0.832 (0.702, 0.865)
**eighth week**	Tran	82.21 (72.72–99.02)	83.22 (69.80, 96.40)	77.08 (59.96, 96.35)	74.60 (61.93, 87.26)	84.89 (72.51, 97.28)	0.855 (0.706, 0.938)
	Sag	81.15 (71.03–95.58)	81.38 (68.58, 94.04)	79.40 (62.58, 97.04)	77.94 (66.58, 89.31)	82.02 (68.95, 95.09)	0.838 (0.694, 0.929)
	Tran+ Sag	77.88 (77.14–95.19)	77.55 (72.13, 95.19)	82.94 (75.59, 93.01)	84.48 (74.75, 94.22)	75.56 (67.66, 83.46)	0.858 (0.729, 0.829)
**Overall**	Tran	79.34 (73.44, 85.25)	77.06 (67.42, 86.70)	81.05 (73.89, 88.21)	82.22 (77.63, 86.82)	74.82 (59.65, 89.99)	0.843 (0.801, 0.884)
	Sag	80.07 (75.49, 84.66)	79.18 (72.63, 85.72)	81.36 (75.13, 87.60)	81.85 (77.82, 85.88)	78.21 (69.03, 87.38)	0.834 (0.776, 0.893)
	Tran+ Sag	80.32 (72.38, 88.25)	80.73 (69.16, 92.30)	80.91 (76.75, 85.08)	81.18 (79.10, 83.26)	80.02 (66.37, 93.68)	0.857 (0.793, 0.922)

Overall: Include all data for 6–8 weeks

*Auc* Area Under Curve, *CI* Confidence Interval, *Tran* Transverse plane, *Sag* Sagittal plane, *PPV* positive predictive value, *NPV* negative predictive value

#### Comparison of different gestational weeks and different planes

**Fig. 6:**
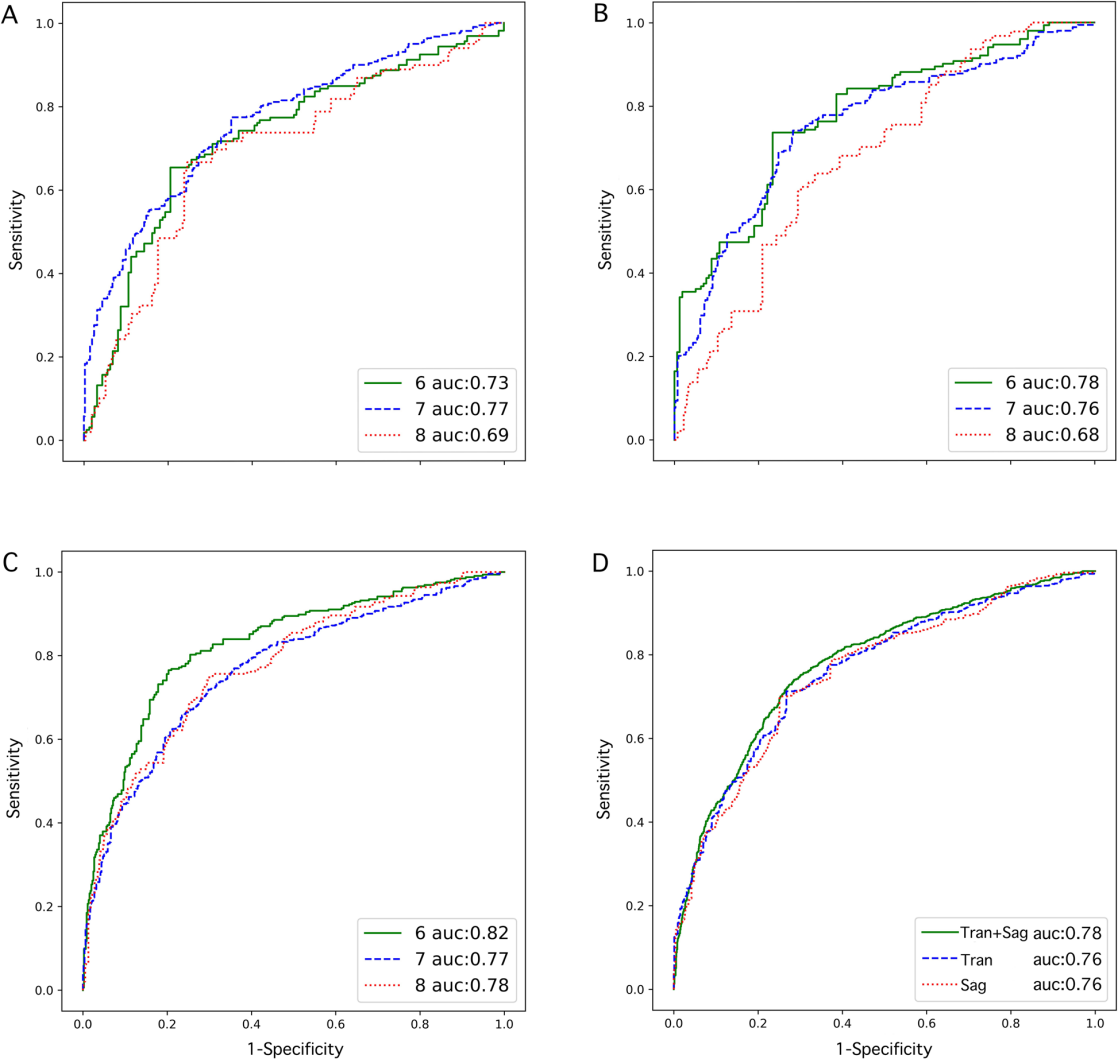
Receiver operating characteristic curves of the deep convolutional neural network model for prediction of miscarriage in the retrospective study. **A** The transverse plane of different gestational weeks; **B** The sagittal plane of different gestational weeks; **C** The sagittal combined transverse plane of different gestational weeks. **D** The overall gestational weeks of different planes. Tran: Transverse plane; Sag: Sagittal plane

To be analyzed by different gestational weeks, the sixth week had a better classification effect, as the AUC for the sixth week was 0.904 (95% confidence interval [CI], 0.781–0.950), which was higher than the other 2 groups (seventh week, 0.832 (95%CI, 0.702–0.865); eighth week, 0.858 (95%CI, 0.729–0.829)). We also analyzed different ultrasound planes; the AUC of the sagittal combined with transverse planes was 0.857 (95%CI, 0.793–0.922), which showed better diagnostic performance compared with the sagittal (AUC, 0.843 (95%CI, 0.801–0.884)) or transverse plane (AUC, 0.834 (95%CI, 0.776–0.893)) alone. The ROC curves were shown in Fig. 6.

#### Prospective study

In order to verify the actual diagnostic ability of our model, we used prospective data as the test set, based on the model trained by the previous retrospective data. Because the sagittal combined with transverse planes showed better diagnostic performance compared with the sagittal or transverse plane alone, we used the sagittal combined with transverse planes for analysis in the prospective study. It had good predictive ability in predicting early pregnancy miscarriage, with the accuracy, sensitivity, specificity, PPV, and NPV of 78.10, 80.39, 94.52, 94.89, and 77%, respectively, and an AUC of 0.885(95%CI, 0.846–0.925) (Table 3).

**Table 3:** Classification results of the prospective study

Methods	Characteristics	Accuracy,% (95%CI)	Sensitivity,% (95%CI)	Specificity,% (95%CI)	PPV,% (95%CI)	NPV,% (95%CI)	AUC (95%CI)
**CNN**	VGG19	78.10 (76.21, 79.99)	80.39 (78.59, 82.18)	94.52 (89.15, 99.88)	94.89 (89.85, 99.88)	77.00(75.27, 78.72)	0.885 (0.846, 0.925)
**ultrasound characteristics**	CRL	66.34 (56.65, 74.82)	66.67 (29.57, 90.75)	66.32 (56.32, 75.04)	11.11 (3.82, 25.91)	96.92 (88.83, 99.78)	0.665 (0.564–0.756)
	HR	82.18 (73.49, 88.51)	50.00 (18.76, 81.24)	84.21 (75.46, 90.31)	16.67 (5.01, 40.05)	96.39 (89.47, 99.20)	0.671 (0.570–0.761)
	CRL + HR	83.17 (74.59, 89.31)	50.00 (18.76, 81.24)	87.37 (79.06, 92.77)	20.00 (6.28, 45.95)	96.51(89.82, 99.23)	0.687 (0.587–0.775)

VGG19: a model of the convolutional neural network

*Auc* Area Under Curve, *CI* Confidence Interval, *CNN* convolutional neural networks, *CRL* crown-rump length, *HR* heart rate, *PPV* positive predictive value, *NPV* negative predictive value

#### Comparison with the manual ultrasound characteristics

**Fig. 7:**
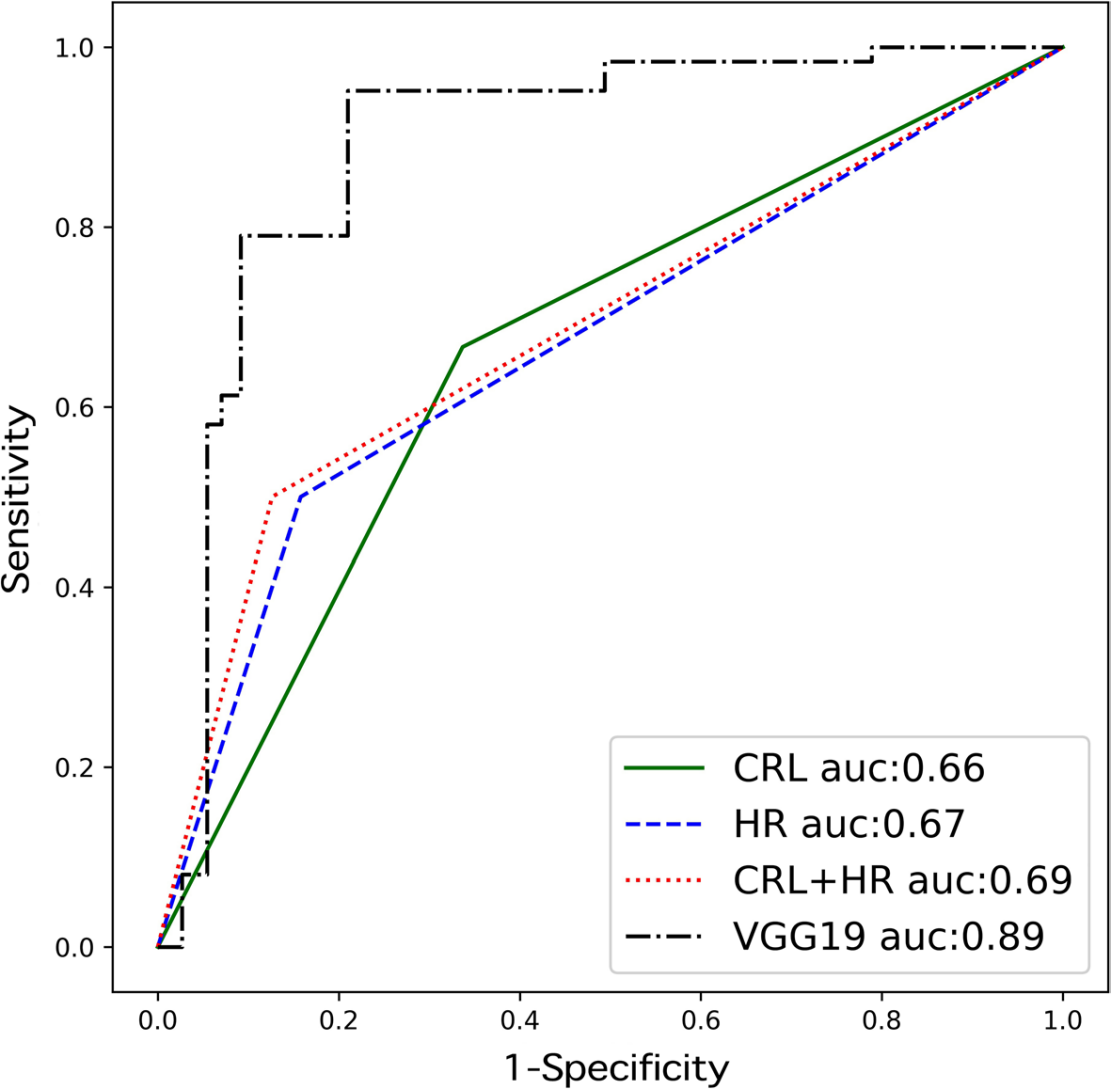
Receiver operating characteristic curves of the CNN model and Ultrasound indexes in the prospective study. CRL: crown-rump length; HR: heart rate; CNN: convolutional neural network. VGG19: a model of the convolutional neural network

We analyzed the fetal heart rate and crown-rump length according to the research of DeVilbiss et al. [5] using our prospective data, and we obtained various indicators of diagnosis (Table 3). The predictive power of the CNN for reviewing ultrasound characteristics was higher compared with performing manual measurements of ultrasound features (the AUC was 0.885vs 0.687, respectively) (Fig. 7).

## Discussion

In the retrospective study, we evaluated the feasibility of CNN-based deep learning algorithms for the prediction analysis of miscarriage in early pregnancy ultrasound images of the gestational sac. The best-performing model yielded satisfactory predictions on the test set, with an AUC of 0.857, a sensitivity of 80.73%, and a specificity of 80.91%. For the prospective study, the sensitivity and specificity of the classification were 80.39 and 94.52%, respectively, and the AUC was 0.885. This work suggests an improved approach to the assessment of the early pregnancy sac. Our research focused on pregnancies with a detectable fetal heart rate, instead of pregnancies without fetal cardiac activity, the clinical prediction of low-risk pregnancies was more practical. According to the prediction model, clinicians can provide better consultation and service for pregnant women, make a more reasonable and humanized follow-up plan, shorten the examination cycle and pay attention to the embryonic development status of the pregnant women at risk of miscarriage.

Most existing studies investigate the individual components of the first-trimester ultrasound for predicting miscarriage [9, 25, 26]. Abnormal yolk sac size and appearance have been reported to be useful markers for miscarriage prediction before the demonstration of fetal viability [27]. However, in presence of an established viable intrauterine pregnancy, its usefulness is limited. Pillai et al reported that the crown-rump length has a lower predictive value than the fetal heart rate [28]. This could be due to the fact that the measurement of the crown-rump length in early pregnancy is affected by inter-observer variation [29]. Some studies used 3D ultrasound to observe the volume of the gestational sac or trophoblast to predict early pregnancy miscarriage [30–32]. However, the post-processing of volume data is a lengthy process, which limits its clinical application. Gestational age also needs to be considered when using these sonographic markers, because they change with the gestational age throughout the first trimester. However, for spontaneous conception, the exact gestational age cannot be determined in women whose last menstrual period was uncertain or whose menstrual cycle was irregular [33]. Even in those with known last menstrual period and regular cycles, there is a discrepancy of more than 5 days in gestation calculated from the menstrual history and by ultrasound in about 25% of cases [34]. The miscarriage prediction mode we had made by CNNs focused on the morphologic characteristics of the gestational sac, gestational age was not considered as an analysis parameter. Although the ultrasound images of the sixth week performed better at predicting miscarriage, which may have been related to the morphologic changes of the gestational sac during embryonic development, the seventh and eighth weeks also have good prediction results. Recently, DeVilbiss et al. [5] found that low fetal heart rate and small crown-rump length were independent predictors of clinical pregnancy loss, with the greatest risks observed for pregnancies having both characteristics. In order to better illustrate the potential predictive power of our model, we also compared with the manual ultrasound measurements in a prospective study. The results suggest that our CNN method is fast, convenient, and provides more accurate results.

To our knowledge, this is the first study to analyze ultrasound image features using CNN to predict the subsequent risk of pregnancy loss during early pregnancy. In our research, machine learning makes up for the shortcomings of intra-observer and inter-observer variation of manual ultrasound measurements in the prediction of early pregnancy miscarriage. Because machine learning is automatic from extraction to calculation, it evaluates ultrasound images more quickly and objectively. At the same time, it can quantitatively calculate the characteristics of ultrasound images, which is most beneficial for the classification of data. We have not only established a predictive model through a large number of cases in a retrospective study, but we also conducted prospective experiments to verify the predictive ability of the model, so that it can be better applied to clinical practice in the future.

Pregnant women, particularly those who have recurrent miscarriages, have anxiety and fear regarding the potential for miscarriage during early pregnancy. Antenatal depression and anxiety impact fetal development and seem to have a long-term detrimental effect on children’s mental health [7, 35]. It suggests that in addition to the medical value of the ultrasound, it also has an important psychological value that has to be considered in order to guarantee integral care of the pregnant women, especially in the first trimester [36]. Our study tried to predict the possibility of future miscarriage at the time of the first appearance of the fetal heartbeat, it would be useful to obstetricians for counseling their patients, relieving their tension and anxiety, and guiding the short-term management of these pregnancies. A further clinical application of the predictive model would be to equip ultrasound machines with machine-learning capabilities to provide the probability of miscarriage by one click, assist clinicians in patient consultation.

Our research surpasses the limitations of manual measurement of clinical ultrasound image parameters and calculates the image features quantitatively and automatically. In addition, we found that the prediction ability of the transverse plane combined with the sagittal plane was better than that of a single plane, indicating that the scanning of multiple planes provides more comprehensive information. Our method is simple, fast, and more suitable for clinical application, which can make clinicians more confident in their diagnosis and consultation.

However, there are still many limitations in our study. The performance of CNNs is dependent on the images that are fed to the algorithm. The higher resolution ultrasound images performed by experienced sonographers will aid in the improved predictions. In the retrospective study, most of the images we included had irremovable measurement marks, which may explain why the deep learning algorithm cannot achieve a better learning performance. But on the other hand, it also shows that our method is more robust, has lower requirements on input prediction images, and is more suitable for clinical diagnosis. In addition, the number of prospective cases is small, more cases will be used to train the model, and a multicenter, large-scale prospective study, such as a large birth cohort study, will be conducted in the future. In addition, our model only considers the characteristics of the ultrasound image in two-dimensional. In the future, we would try to analyze the 3D ultrasound volume data of the gestational sac combined with other ultrasound and biochemical markers to optimize a better prediction model.

## Conclusion

The CNN is feasible to predict miscarriage by identifying the morphologic characteristics of the gestational sac in early pregnancy, and it can be used clinically in the future to provide assistance during clinician-patient consultations.

## Supplementary Information

**Additional file 1: Figure S1.** The architecture diagram of VGG19 model.

## Data Availability

The datasets used and/or analyzed during the current study are available from the corresponding author on reasonable request.

## Abbreviations

AUC: Area under the receiver operating characteristic curve
CI: Confidence interval
CNN: Convolutional neural network
FN: False negative
FP: False positive
GT: Gold standard
NPV: Negative predictive value
PPV: Positive predictive value
ROC: Receiver operating characteristic
TP: True positive
US: Ultrasonography
