# Supplementary figures and images for: Automated prediction of early spontaneous miscarriage based on the analyzing ultrasonographic gestational sac imaging by the convolutional neural network: a case-control and cohort study
Source: BMC Pregnancy Childbirth. 2022 Aug 5;22:621. doi: 10.1186/s12884-022-04936-0 (PMC9354356; doi:10.1186/s12884-022-04936-0)

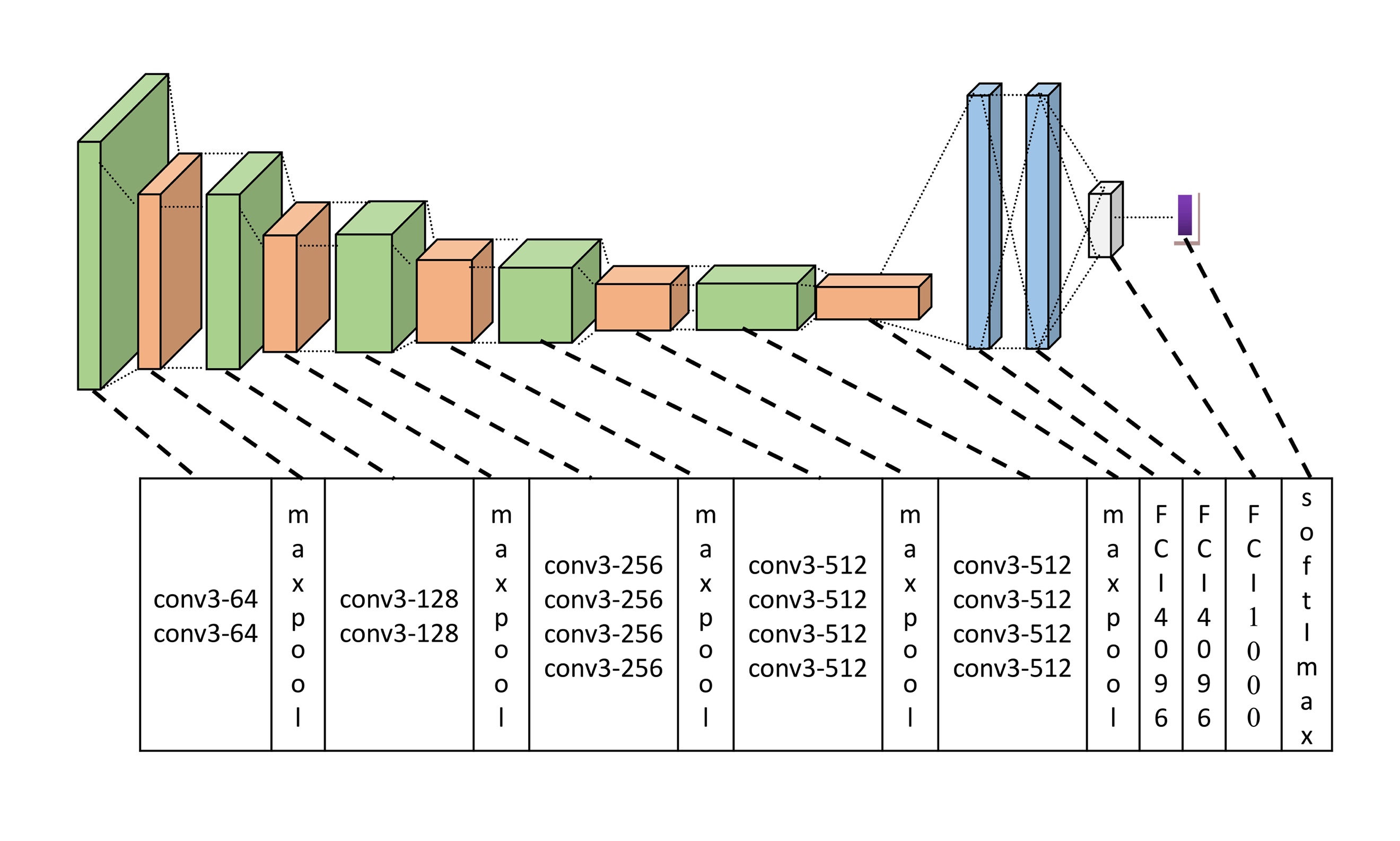


Figure S1. The architecture diagram of VGG19 model.

Supplement: Supplementary file 1 — Additional file 1: Figure S1. The architecture diagram of VGG19 model. [file 12884_2022_4936_MOESM1_ESM.docx]
